# Obesity on Campus

**Published:** 2007-06-15

**Authors:** Phillip B Sparling

**Affiliations:** School of Applied Physiology, Georgia Institute of Technology

The "O" word on campus is not *Oprah* or *online;* it is *obesity*. During the past few years, administrators and faculty members have been discussing obesity as they see greater numbers of overweight students. News media accounts about rising rates of obesity provide an impersonal perspective of the problem, but observing obesity face-to-face in our classrooms and across our campuses makes it real. Increasing numbers of "super-sized" students are right in front of our eyes. They should not be ignored.

Epidemiologists paint a gloomy picture of the health of our young people. During the past two decades, a significant increase in obesity and obesity-related disorders such as type 2 diabetes, hypertension, and dyslipidemia has occurred among people in their teens and 20s (1,2). Those of us who are middle-aged have difficulty accepting chronic disease in our own lives; seeing it prematurely affect the younger generation is even more distressing.

Little progress has been made in controlling or preventing obesity, because the cards are stacked against us. Obesity is a complex adaptation to existing environments that greatly favor high energy intake and low energy expenditure (3). Food is everywhere, and it is generally inexpensive, flavorful, large-portioned, and high-calorie. In addition, we rely on energy-saving devices and technology throughout the day, and most of our waking hours are spent sitting.

No medical breakthrough is on the horizon. Even with continued advances in genomics and molecular medicine, scientists are unlikely to discover an effective, safe, and affordable drug that would cure or prevent obesity. From a public health perspective, the best solution remains encouraging positive behavior changes associated with diet and physical activity. However, although obesity is generally acknowledged as a serious problem and difficult to solve, many college and university leaders view helping overweight students as being outside the purview of higher education.

## Recognizing the Need for Action

Concern about student behaviors related to alcohol, drugs, and sexuality overshadows the need to emphasize the mundane but important habits of eating healthfully and exercising regularly. Campus student services have long been established to manage the perennial coming-of-age issues associated with substance abuse and sexual behavior. These programs are continually updated to guide students toward responsible decision making and to minimize crises. Let the good work continue. However, excess body weight has emerged as a new challenge. The data are convincing. Findings from a national survey conducted in 2005 indicate that 3 of 10 college students are either overweight (body mass index [BMI] 25.0–29.9 kg/m^2^) or obese (BMI ≥30.0 kg/m^2^) (4). Behaviorally, 9 of 10 students eat fewer than five servings of fruits and vegetables per day, and nearly 6 of 10 students participate fewer than 3 days per week in vigorous-intensity (20 minutes or more) or moderate-intensity (30 minutes or more) physical activity (4). Although excess weight, poor nutrition, and insufficient physical activity are not emergencies that require immediate action, their urgency should not be underestimated.

The predominant way obesity is thought about and confronted — particularly at research universities — is far removed from the current needs of overweight students. The emphasis is on scientific inquiry; researchers focus on unraveling the biological complexities of obesity and related medical conditions. Although universities assemble top-notch biomedical teams to conduct basic research, the institutions often ignore the problem of obesity on their own campuses. This type of disconnect is not new to academia, and the explanation is straightforward. Universities are increasingly dependent on external dollars to remain solvent, and eminent research teams bring in major funding from federal agencies and biotechnical companies.

Simply put, a significant gap persists between the quest for new clues to solve the obesity puzzle (basic biomedical research) and the application of current evidence-based practices to prevent and treat obesity now (applied behavioral research). The good news is that funding is becoming available to study how to improve the translation process at all levels. By better understanding the barriers to transfer and by focusing on context and connectedness, we can find ways to accelerate the translation of evidence-based recommendations into practice and policy (5).

Another problem is that obesity is an emotionally charged issue surrounded by prejudices and misconceptions. We are prone to judge obesity as a personal failing or a sign of insufficient will power — perceptions that are almost always incorrect. People don't choose to be obese. Many paths lead to obesity, and each is shaped by a unique combination of intertwined biological, psychosocial, environmental, and cultural influences. Moreover, although the term *obesity* has a specific meaning to researchers, clinicians, and educators, it is thought to be pejorative by many students, patients, and the public. We must be sensitive to this belief and, depending on the target audience, take the time to more clearly define the term or use alternative language such as *overweight* or *excess weight*.

The awkwardness of discussing obesity should not deter us from addressing the problem. Colleges and universities have long dealt with complex, controversial issues. Enlightenment is our business: we should be educating all of our students — obese or not — about issues related to diet, exercise, and behavioral change. We should openly discuss the stigma of obesity and how we can combat it. Resources to help students reach and maintain a healthy weight can be provided in a sensitive, nonjudgmental, and professional manner. Many students would be grateful for such services.

## Meeting the Challenge

We have a special opportunity to tackle the problem of obesity on our campuses. For many young people, college is the first major step toward independence and charting their own courses. Students are open to change and challenge as they reexamine their lifestyle choices. We can capitalize on this ripe-to-change period by encouraging students to improve eating and exercise habits and teaching them how to implement healthful changes. At the same time, we have the capacity to provide a supportive environment by providing and promoting good food choices and multiple options for physical activity, including pedestrian-friendly campuses.

The goals are to decrease the prevalence of obesity through healthy weight loss and maintenance of weight loss among the overweight and to prevent excess weight gain among the nonoverweight. To date, weight management efforts on college campuses have been fragmented. Just as the causes of obesity are multifactorial, solutions must be both broad in scope and coordinated. A long-term plan to deal with student obesity can build on existing programs and need not be expensive. However, administrators must support and even champion the cause. Logical steps include establishing a campuswide team that represents key campus units, such as the president's office, campus planning, campus recreation, the counseling center, food services, student health, student government, and selected academic departments; taking stock of current resources and policies; coordinating efforts and developing realistic goals and strategies for reaching them; and following through by implementing a healthy weight initiative, monitoring progress, and adjusting accordingly.

Examples of programs include instituting an across-the-curriculum course on healthy weight with information on nutrition, physical activity, energy balance, and self-management skills; hosting a semiannual speaking engagement with a prominent health or medical authority; reviving or expanding physical education and fitness classes to expose students to a variety of enjoyable, potentially lifelong activities (6); developing and promoting walking and cycling routes on campus (7); and renegotiating contracts with campus food vendors for healthier fare. When well-known U.S.-based companies, including Wendy's International, Inc and the KFC Corporation, as well as New York City, are eliminating *trans* fats from their restaurants' menus, surely colleges can be bolder and hold vendors to higher standards for healthier foods (8). Student health centers should offer weight management services to overweight students. University obesity clinics do not meet this need, because most are based only in medical schools and either offer fee-based programs open to the community or are research-focused and have participant selection criteria. To promote a campuswide healthy weight initiative, popular professors, coaches, students, and local luminaries could be recruited as spokespeople.

More than ever, consensus exists among professionals (e.g., scientific and medical organizations, public health agencies) on the key lifestyle recommendations related to physical activity, diet, and healthy weight (9-13). Evidence for the synergistic benefits of regular exercise and good nutrition in both primary and secondary prevention across multiple chronic diseases is unambiguous and continues to grow. Our health messages to Americans have never been clearer or stronger.

Colleges will evolve different strategies that make sense for their students and campuses. The eventual aim is to establish a unified campuswide team that can efficiently coordinate and creatively promote classroom, health care, and extracurricular programs that target obesity. Solutions should be sustainable and manifest in food services, in building and campus design, and through policy (14,15). Campuswide approaches are more likely to succeed, because competing interests and barriers can be identified early. Programs that require changes in curricula, policies, campus environments, or business operations hold the greatest promise for making a difference but also pose the greatest challenge. Strategic thinking and political aplomb are important ingredients for success. A necessary precondition, however, is institutional commitment.

## Action Needed Sooner Not Later

Remember that the mission of higher education is to develop the total person. Check your college catalog: along with the term *well-educated*, terms such as *ethical*, *civic-minded*, *industrious*, and *healthy* are used to describe the ideal graduate. Although these attributes correlate with high standards, helping students achieve them commits us to goals that many of us still hold dear and strive to reach in our various university roles. In that spirit, colleges should be sensitive to the broader needs of their students and should respond appropriately as those needs change.

We must recognize that obesity is a real threat that compromises the overall well-being and development of our students. Much is at stake — these young people are not just our future alumni but America's next generation of leaders. Colleges need to step forward, develop an action plan, and follow through. Coordinated campuswide strategies that focus on healthy eating, regular physical activity, and effective weight management can benefit all members of the campus community. The vision is to make healthy decisions easier, more convenient, and enjoyable through supportive environmental changes and forward-thinking policies.

Academics value the world of ideas, believe in social causes, and relish the opportunity to solve real-world problems. Obesity is a problem whose deleterious effects are occurring not just in our world but on our campuses. We do have a vested interest. And who is better positioned and more able to look for its solutions than we are? Let's rally the forces to meet this challenge now.
